# Combination of strategies to initiate clozapine for refractory schizophrenia in a patient with low neutrophil levels

**DOI:** 10.1136/bcr-2021-247734

**Published:** 2022-10-13

**Authors:** Keisuke Takanobu, Naoki Hashimoto, Shuhei Ishikawa, Ichiro Kusumi

**Affiliations:** 1 Forensic Psychiatry Center, Hokkaido University Hospital, Sapporo, Hokkaido, Japan; 2 Department of Psychiatry, Hokkaido University Graduate School of Medicine, Sapporo, Hokkaido, Japan

**Keywords:** Psychiatry (drugs and medicines), Schizophrenia, Drugs: psychiatry, Haematology (incl blood transfusion)

## Abstract

Clozapine is the only drug with confirmed efficacy for refractory schizophrenia; however, its use is restricted due to the risk of potentially life-threatening side effects, such as agranulocytosis. Although this restriction ensures safety against haematological risks, some patients with refractory schizophrenia who have low neutrophil levels may miss the opportunity to receive clozapine treatment. We herein report the case of a patient with refractory schizophrenia and low neutrophil levels who was successfully initiated on clozapine treatment after the use of several methods for increasing neutrophil levels. These strategies consisted of discontinuation of antipsychotics, treatment with lithium carbonate and adenine, and light exercise before blood testing. Combining these procedures may be an effective option in the treatment of patients with refractory schizophrenia whose neutrophil levels are not sufficient to initiate clozapine.

## Background

Clozapine is an effective drug for refractory schizophrenia; however, its use is restricted due to the risk of potentially life-threatening side effects, such as agranulocytosis.^1^ Low baseline levels of leucocytes and neutrophils are risk factors for clozapine-induced neutropenia and agranulocytosis.^2^ In most countries, mandatory haematological monitoring and strict regulations have been implemented to reduce these haematological risks. In Japan, patients with schizophrenia who are prescribed clozapine must register with the Clozaril Patient Monitoring Service for patient management.^3^

In Japan, a common clinical problem is that clozapine cannot be initiated in patients whose baseline leucocyte and neutrophil counts are ≤4.0×10^9^/L and ≤2.0×10^9^/L, respectively. This restriction is more stringent in Japan than in other countries.^4–6^ In other countries, considerations have been established for patients with low white blood cell counts and absolute neutrophil counts (ANCs) due to benign ethnic neutropenia (BEN), and clozapine treatment may be initiated after receiving consent from a haematologist.^4 5^ In addition, patients with BEN are managed according to a different ANC algorithm with relaxed criteria for baseline ANC levels.^4 5^ However, this consideration has not been established in Japan.^5^ Therefore, it is expected that more people in Japan than in other countries will miss the opportunity to receive clozapine due to low leucocyte and neutrophil levels. Clozapine is the only drug that is confirmed to be effective for refractory schizophrenia; thus, those denied clozapine as a treatment option are at a significant disadvantage.

In this case report, a patient with refractory schizophrenia who had earlier been denied clozapine due to low neutrophil levels was successfully administered clozapine following a combination of several strategies to increase neutrophil levels.

## Case presentation

**Figure 1:**
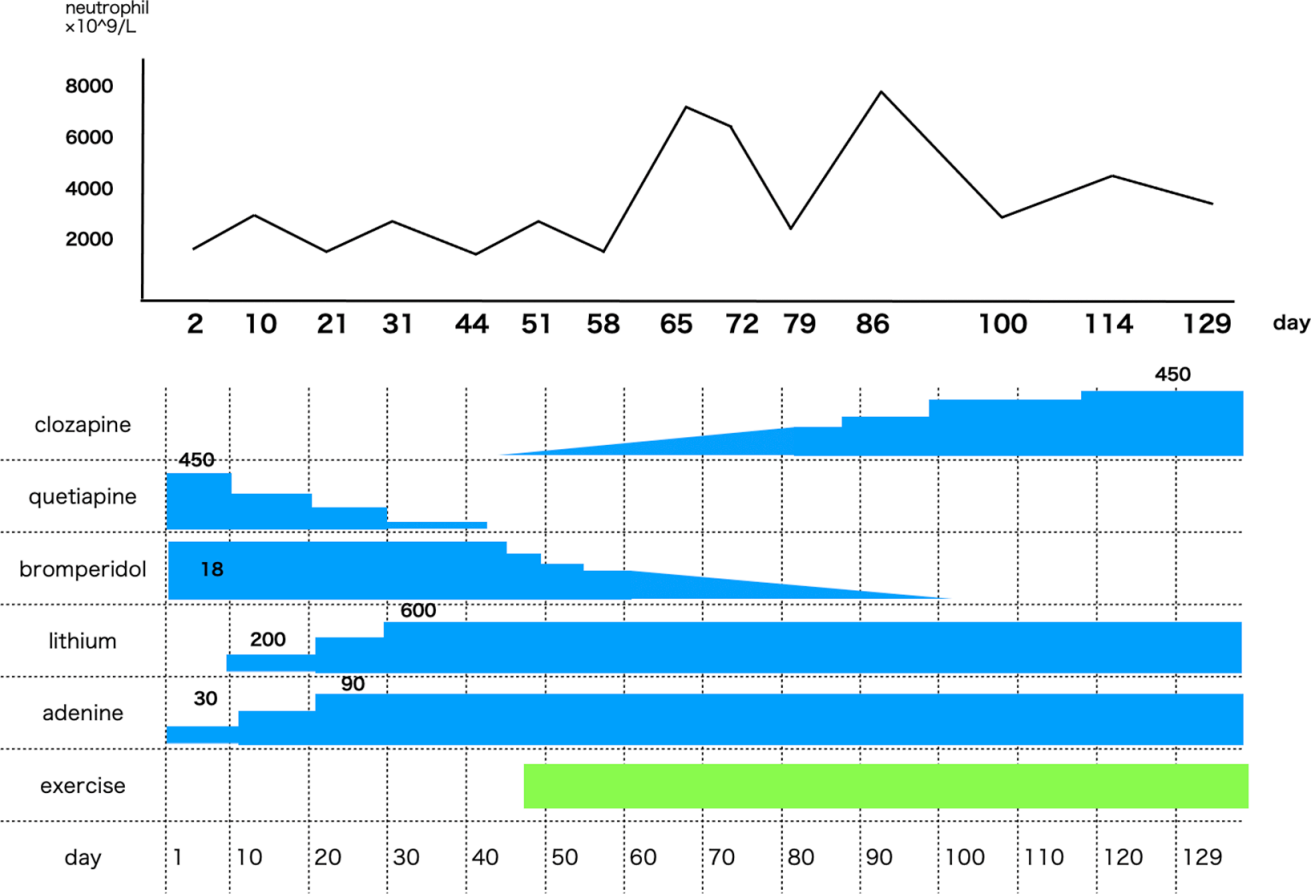
Neutrophil counts and treatment course over time.

A man in his 40s had been experiencing symptoms of paranoid schizophrenia from his late 20s. He had been diagnosed with schizophrenia 13 years previously and was started on antipsychotic medication. Several antipsychotics (including risperidone, aripiprazole and olanzapine) were prescribed; however, all were discontinued due to side effects or poor treatment efficacy owing to his thought disorder. Administration of clozapine had been considered 5 years previously; however, it was abandoned due to a low neutrophil count (1.8×10^9^/L). As there was no further improvement in his psychiatric symptoms, he was admitted to the hospital for clozapine initiation (day 1). The Brief Psychiatric Rating Scale (BPRS) score on admission was 49 points (moderate). Treatment strategies and neutrophil counts over time are shown in figure 1. Blood tests at admission showed a leucocyte count of 4.9×10^9^/L and a neutrophil count of 1.6×10^9^/L. The neutrophil level was below the lower limit for clozapine initiation. The haematologist ruled out haematological disorders and vitamin B_12_ and folate deficiency as the possible causes for neutropenia.

Since there was no organic cause for the neutropenia, we planned a treatment course to improve the neutrophil count.

To improve the low neutrophil count, a combination of four strategies was implemented: (1) reduction or discontinuation of antipsychotics, (2) treatment with lithium carbonate, (3) treatment with adenine and (4) light exercise before blood testing.

## Treatment

Due to his severe psychiatric symptoms, we decided to start reducing the dosage of quetiapine while continuing the main medication, bromperidol. Quetiapine (450 mg on admission) was gradually reduced from day 1 and discontinued on day 42. Adenine was started on day 1, and the dose was increased from 30 mg to 90 mg by day 29. Lithium carbonate was started on day 7 as a dose of once a day and increased from 200 mg to 600 mg/day on day 29. The blood lithium level on day 32 was 0.66 mEq/L. Clozapine was initiated on day 44 when the neutrophil count was 2.8×10^9^/L. However, the neutrophil level remained at the lower limit during clozapine initiation. On day 44, light exercise (15 min of exercise and a walk) was also initiated before the blood test. In addition, dose reduction of bromperidol (18 mg at the time of admission) was initiated simultaneously with the initiation of clozapine, and bromperidol was discontinued on day 104.

## Outcome and follow-up

As a result of these strategies, the neutrophil count increased to 5.5 × 10^9^/L on day 65, and clozapine administration was continued. The clozapine dose was subsequently increased to 450 mg by day 114. The patient was discharged on day 129. The BPRS score on discharge was 46 points (moderate). Following discharge, no neutropenia was observed, and the patient continued clozapine treatment for >2 years.

## Discussion

In this report, we describe the improvement of long-term low neutrophil levels and successful initiation of clozapine by a combination of four strategies: (1) discontinuation of antipsychotics, (2) treatment with lithium carbonate, (3) treatment with adenine and (4) light exercise before blood testing.

First, with regard to the discontinuation of antipsychotics, several drugs, including antipsychotic drugs, have been reported to increase the risk of neutropenia or agranulocytosis in patients treated with clozapine.^7 8^ Although the cause of drug-induced agranulocytosis has not been fully elucidated, direct toxicity and immune mechanisms have been speculated. Direct toxicity to myeloid cells has been reported to be associated with the use of antipsychotic drugs, antiepileptic drugs and non-steroidal anti-inflammatory drugs.^7^ In addition, antibacterial drugs, autonomic agents and gastrointestinal drugs (such as proton pump inhibitors) may worsen the haematological side effects of clozapine when administered concomitantly.^8^ The effects of these concomitant medications should always be considered if the neutrophil level is low.

Second, lithium carbonate causes reversible leucocytosis, which may ameliorate low neutrophil levels. Although the mechanism by which lithium increases the number of neutrophils is not fully clear,^9^ it is hypothesised to be related to the increased production of granulocytes via the enhanced action of colony-stimulating factor on colony-forming cells.^10–13^ Moreover, lithium causes both acute and chronic increases in leucocytes and neutrophils^10^ and has been used to increase neutrophil counts in patients who have developed neutropenia while on clozapine.^11–13^ In addition, the dosage of lithium carbonate can affect the leucocyte-increasing effect. Although no clear dose dependence of the leucocytopoietic effect has been demonstrated, a blood lithium level of ≥0.4 mEq/L is required for this effect.^14–16^ Many case reports showing efficacy of lithium carbonate for clozapine-induced neutropenia have blood levels above 0.6 mEq/L.^16–18^ Furthermore, the incidence of leucocytosis is higher with administration of once a day than with two times a day.^19^ Therefore, in this case, the dosage of lithium carbonate was set to once a day and titrated up until it exceeded 0.6 mEq/L, resulting in a dose of 600 mg.

Third, adenine is reportedly effective in the treatment of radiation-induced and drug-induced leucopenia.^19 20^ The mechanism underlying the effect of adenine in leucopenia is unclear, though it is considered to be absorbed by RNA/DNA in the bone marrow and used for nucleic acid synthesis.^21^ Moreover, adenine decreases the frequency of treatment discontinuation due to clozapine-induced neutropenia.^11^ Given the proposed mechanisms of adenine, it is expected to act synergistically with other means for increasing leucocyte counts.

Fourth, exercise evokes leucocyte recruitment by mobilising leucocytes into the blood circulation from the marginated pool in the blood vessels and bone marrow.^22–24^ In situations such as immediately after an overnight bed rest, benign neutropenia may occur because neutrophils are attached to the vascular endothelium.^25 26^ Considering this physiological mechanism, exercise before blood testing is expected to be effective in leucocyte recruitment.

Finally, even though not adopted in the present case, granulocyte colony-stimulating factor has been used in previous similar cases. Although several reports have shown the effectiveness of granulocyte colony-stimulating factor administration to patients with clozapine-induced neutropenia, most are reports on rechallenges after clozapine-induced neutropenia.^27 28^

With regard to our four strategies, discontinuation of antipsychotics or treatment with lithium and adenine was shown to be effective in increasing neutrophil counts in patients treated with clozapine. However, these strategies alone were not sufficient to increase the neutrophil counts in the current case; due to clinical necessity, we attempted multiple methods simultaneously and parallelly. Although it is difficult to identify which measures were primarily effective, this is the first case in which exercise was observed to have increased the neutrophil count in a patient being administered clozapine. Furthermore, this is the first report to show the additive effect of combining these strategies. Considering that the neutrophil counts were higher and less variable after the initiation of exercise and discontinuation of bromperidol than after the initiation of adenine and lithium carbonate, it may be important to implement a combination of multiple strategies. It should be noted, however, that it is unclear whether this strategy will maintain neutrophil counts in the long term after clozapine induction, and continued monitoring will be required in the future. As clozapine is the only antipsychotic that has been confirmed to be effective in the treatment of refractory schizophrenia, it is clinically crucial to ensure that more patients can benefit from safe administration of clozapine. Thus, further research and longer-term follow-up are needed to determine safe strategies for drug administration and monitoring.

**PATIENT’S PERSPECTIVE**

I have experienced the symptoms of my thought disorder for many years, and I am glad that I was able to receive clozapine because clozapine treatment has the potential to improve my symptoms. I had given up on the chance of receiving it due to my low white blood cell count; therefore, I was happy that I was able to take on the challenge. Clozapine feels right for me. The symptoms of my mood and thought disorder seem to be more tolerable than before.

**Learning points**

- Some patients with refractory schizophrenia cannot be safely treated with clozapine due to low neutrophil levels.
- Strategies to increase neutrophil levels include discontinuation of drugs that may cause neutropenia, treatment with lithium carbonate and adenine, and exercise before blood tests.
- The combination of these methods may have an additive effect on increasing neutrophil levels, but further research is needed to understand these effects and long-term safety.
